# Ruxolitinib found to cause eyelash growth: a case report

**DOI:** 10.1186/s13256-017-1304-5

**Published:** 2017-07-12

**Authors:** Julia Song, Alice Song, Trisa Palmares, Michael Song, Harold Song

**Affiliations:** 1 Southern California Eye Physicians & Surgeons, 800 S. Fairmount Ave., #207, Pasadena, CA 91105 USA; 2 Center for Oculofacial & Orbital Surgery, 3771 Katella Ave., #209, Los Alamitos, CA 90720 USA

**Keywords:** Hypereosinophilic syndrome, Ruxolitinib, Alopecia, Madarosis, JAK, Janus kinase

## Abstract

**Background:**

Hypereosinophilic syndrome is a hematologic disorder in which the eosinophils proliferate.

Oral Janus kinase inhibitors are known to be effective treating hypereosinophilic syndrome. Janus kinase inhibitors have also demonstrated efficacy in alopecia. Madarosis is a condition in which the eyelashes are missing or absent and can been seen in alopecia patients.

**Case presentation:**

We present the case of a 77-year-old Asian man who was diagnosed with hypereosinophilic syndrome, refractive to all medications except ruxolitinib. He responded well. It was noted unexpectedly that his eyelashes grew much longer than they were normally.

**Conclusions:**

Previous studies have demonstrated an improvement in alopecia areata, with increased hair growth on the head and eyebrows. This study demonstrates that longer eyelashes may be another effect of oral Janus kinase inhibitors. We report the first case of eyelash elongation and thickening in a patient taking ruxolitinib. Physicians and patients should be aware of the side effect of these Janus kinase inhibitors. Further investigation is needed to ascertain whether ruxolitinib or other interleukin inhibitors can aid in the treatment of madarosis.

## Background

Hypereosinophilic syndrome (HES) is a rare hematologic disorder in which the eosinophils proliferate. The eosinophils infiltrate and damage multiple organs. Without treatment, the condition is fatal, usually due to cardiac dysfunction, particularly left ventricular dysfunction. Causes of reactive eosinophilia include allergic and hypersensitivity reactions, drug allergies, cytokine therapy (recombinant human interleukin), cutaneous disorders, connective tissue diseases, collagen vascular disorders, parasitic infections, immunodeficiency disorders, sarcoidosis, and neoplasms, such as Hodgkin’s and non-Hodgkin’s lymphomas and acute lymphoplastic leukemia. Various hematopoietic neoplasms that demonstrate blood eosinophilis are B cell and T cell non-Hodgkin’s lymphomas, chronic myeloproliferative disorders (chronic: chronic myelogenous leukemia, chronic eosinophilic leukemia, systemic mast cell disease; acute: acute myeomonocytic leukemia (AML) with eosinophilia, acute lymphoblastic leukemia with eosinophilia, and other myeloid leukemias).

Ruxolitinib is approved for myelofibrosis (MF) and is effective in reducing splenomegaly. The medication is off-label for HES but has been reported to be successful in eosinophilic leukemia. Ruxolitinib (Jakafi®) has been reported to aid in refractory alopecia areata. Our patient noted that his eyelashes grew significantly. This is the first case in which eyelash growth has been reported.

## Case presentation

A 77-year-old Asian man presented with sudden onset pruritus. There was no history of drug allergy or scabies. His past medical history was significant for hypertension, benign prostatic hypertrophy, and early glaucoma. His past surgical history included vasectomy, laser iridotomies, and pterygectomies. His medications included losartan, doxazosin and silodosin, amlodipine, carvedilol, and a multivitamin. He was a retired pediatrician who was actively involved in gardening, carpentry, roller-blading, swimming, and walking his dogs. He has had healthy dogs his entire life and currently has dogs.

Our patient first presented to his primary care physician, who offered a psychosomatic explanation for his symptoms. A complete blood count (CBC) with a differential demonstrated elevated eosinophils of 36%. (His white blood cell (WBC) count was 3.7, hemoglobin level was 10.3, platelets were 288, neutrophils 1006, and eosinophils 1314.)

He was referred to an allergist, who started oral prednisone. There was some relief in his symptoms. The dermatologist prescribed interferon. This resulted in extreme fatigue and purple fingernails. There was no improvement in the pruritus, so interferon was discontinued.

He was then referred to a dermatologist who prescribed hydroxyzine and fluocinolone cream. This provided minimal relief. He saw a second dermatologist who performed a skin biopsy, which was negative. Hydroxyurea was prescribed. There was no improvement in the patient’s symptoms or eosinophil count.

Our patient was referred to an oncologist. A bone marrow biopsy (BMB) revealed a slight hypercellularity of 30–40% and increased eosinophilic concentration of 16%. There was no evidence of leukemia, lymphoma, or myelodysplastic syndrome (MDS). Cytogenetic testing revealed the following: 46XY, **del 9 (q13q22)** of unclear significance (as this is seen with AML, but there was no evidence of AML on BMB). His Kappa/Lambda ratio was slightly elevated at 4.15. V-kit Hardy-Zuckerman 4 feline sarcoma viral oncogene homolog (KIT) mutation by polymerase chain reaction (PCR), T cell clonality by PCR, and fluorescence in situ hybridization (FISH) panel results were negative. Immunoglobulin (Ig) testing revealed IgA of 140, IgG of 1317, IgM of 65, and IgE of 358.

Our patient also saw an infectious disease specialist. Human immunodeficiency virus (HIV), antinuclear antibodies (ANA), serum tryptase, lactate dehydrogenase (LDH), vitamin B_12_, urine protein electrophoresis, and parasitic workup (trichinella, toxocara, strongyloides) results were negative. An echocardiogram demonstrated an ejection fraction of 62%.

Our patient continued to have chronic pruritus, resulting in sleepless nights. In desperation, he attempted to discontinue his prostate medications in the hope that his condition was a simple allergy to medication, but this resulted in urinary retention with fever, sepsis, and anasarca, necessitating urgent antibiotic treatment.

Although our patient was negative for the FIP1L1-PDGFRA mutation, a trial of imatinib (tyrosine kinase inhibitor) was given. There was no improvement in his symptoms or condition.

Our patient’s symptoms worsened with continued pruritus and resultant insomnia, so oral prednisone was increased from 20 mg to 80 mg. He noticed no improvement in his symptoms and became clinically depressed.

Additional testing was obtained, revealing the presence of a Janus kinase (JAK) 2 mutation (JAK2 V617F)

He was prescribed ruxolitinib 20 mg orally twice a day. By day 3 of ruxolitinib, he could sleep a full night without pruritus. His prednisone was tapered, and by 1 month, he had tapered off of his prednisone. He developed mild neutropenia, so the dosage of ruxolinitib was decreased. He noted that when he missed even one dose of his ruxolitinib, the pruritus would recur.

His most recent laboratory tests (4 years after his initial presentation and diagnosis of HES) revealed normal eosinophil levels of 2%. He had mild anemia and thrombocytosis, which were stable.

**Fig. 1:**
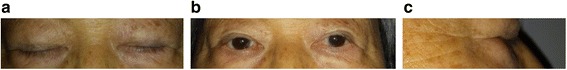
**a** Eyelash growth following ruxolitinib (*front view, eyes closed*), **b** Eyelash growth following ruxolitinib (*front view, eyes open*), **c** Eyelash growth following ruxolitinib (*side view, eyes closed*)

**Fig. 2:**
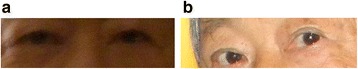
**a** Shorter eyelashes on prednisone daily. Shorter eyelashes on prednisone pulse-dosed every other day. **b** Longer eyelash length, as well as reduced facial edema, was noted with ruxolitinib and discontinuation of prednisone

Several months after starting ruxolitinib, he noted hypertrichosis in both eyes, with increased length and thickness (Fig. 1a,b,c). He did not report additional hair growth on his head, facies, or other parts of his body. He had no history of alopecia throughout his life. He did note that his eyelashes were thinner before starting ruxolitinib (Fig. 2a on prednisone daily and 2b on pulse-dose prednisone).

Unfortunately, due to his tenuous condition, it was decided not to discontinue the oral ruxolitinib in order to measure his pre-medication eyelash lengths.

**Fig. 3:**
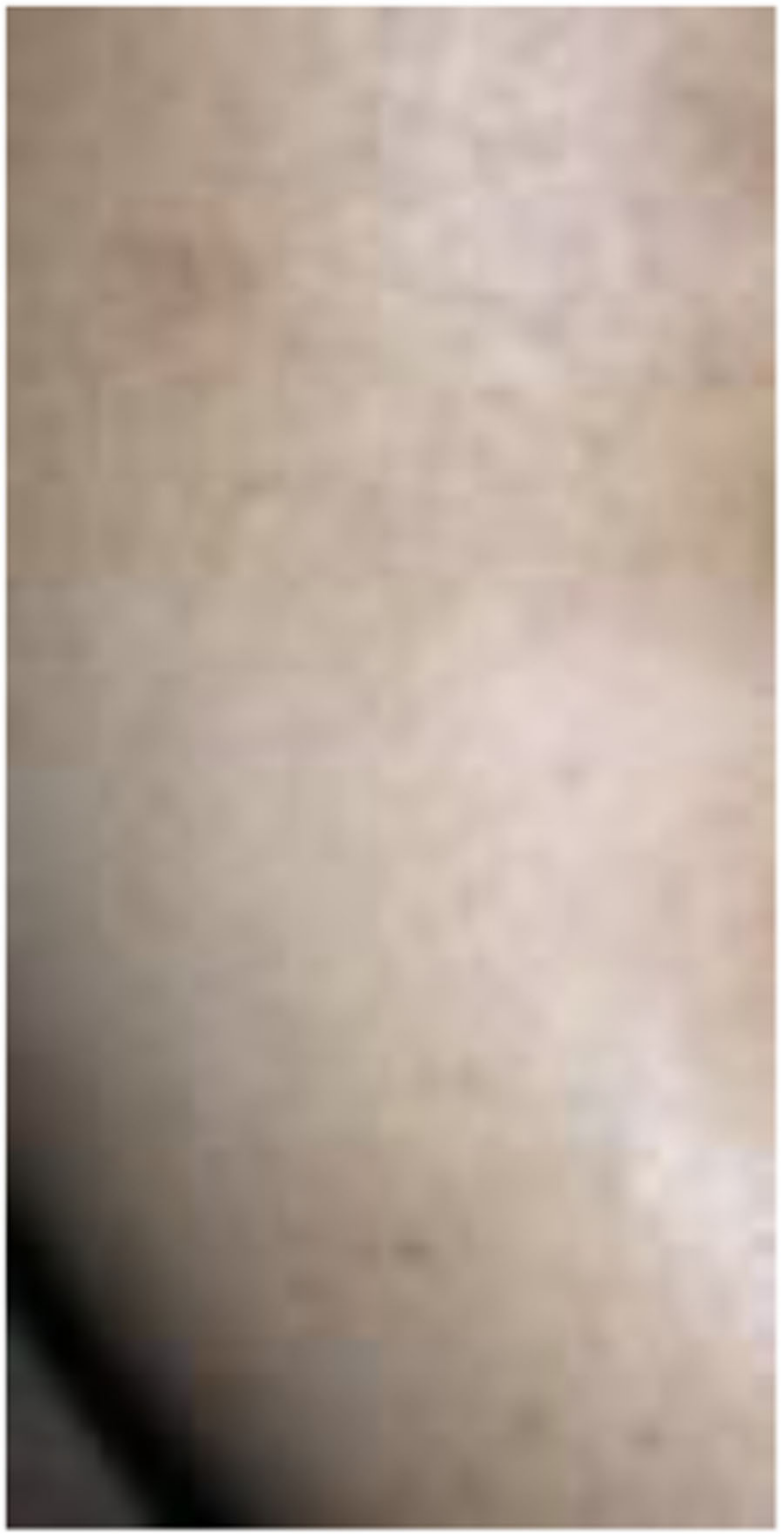
Schwannoma of the left upper extremity developed following ruxolitinib

Our patient developed a painless, mobile, circular lesion in the left upper extremity (0.5 × 0.4 × 0.4 cm) (Fig. 3). A biopsy revealed a schwannoma, containing spindle cells with Verocay bodies with some nuclear pleomorphism. It was resected without complications or recurrence. It is more commonly seen in psoriatic patients.

## Discussion

HES is a potentially fatal hematologic condition that requires prompt diagnosis and treatment. If untreated, patients will succumb to death, usually due to left ventricular cardiac dysfunction. Patients are extremely uncomfortable due to continual pruritus. JAK2 mutations have been identified as a marker and can target specific treatments, particularly ruxolitinib [1–4].

Ruxolitinib (Jakafi®) was approved by the US Food and Drug Administration (FDA) on November 16, 2011, as the first and only treatment for intermediate- or high-risk myelofibrosis (MF) [5]. It has been shown to aid in MF, polycythemia vera, and essential thrombocytopenia, specifically splenomegaly reduction [6].

Ruxolitinib is classified as a biologic response modifier, a signal transduction inhibitor, and a kinase inhibitor. Most specifically, ruxolitinib is a JAK inhibitor. Similar to other kinase inhibitors, ruxolitinib acts intracellularly against a specific signaling pathway. To date, ruxolitinib is the first and only drug approved by the FDA to target the JAK-STAT signaling pathway that involves the JAK family of enzymes. Inhibition of the JAK1 and JAK2 enzymes is ruxolitinib’s mechanism of action. By inhibiting JAK1 and JAK2, ruxolitinib interrupts the signal sent to HPCs that would have resulted in unnecessary hematopoiesis. Therefore, ruxolitinib helps prevent additional marrow fibrosis.

The most common adverse reactions to ruxolitinib are hematologic, specifically thrombocytopenia and anemia, both of which are dose-related. Although neutropenia is less common, thrombocytopenia and anemia (all grades) occurred in 70% and 96% of patients taking ruxolitinib.

Madarosis is a term which was originally coined to denote loss of eyelashes due to destruction of hair follicles, but now encompasses the loss of cilia of both eyelashes and eyebrows [7]. It can occur following trauma, surgery, or chemotherapy. It can be a result of a psychiatric condition, trichotillomania, in which patients have an irresistible urge to pull on their eyelashes.

Madarosis can be divided into two groups: scarring or non-scarring depending upon the etiology. Appropriate diagnosis is essential for management. Follicular unit transplantation has been found to be a useful method of treating scarring madarosis and the procedure relevant to eyebrow and eyelash reconstruction has been discussed.

Madarosis can be disfiguring and cause morbidity. Topical prostaglandins, which have been used in the treatment of glaucoma, have been utilized for eyelash growth for both cosmetic reasons and for medical conditions [8–19]. There was one case of poliosis-induced prostaglandins [12]. Topical prostaglandins may cause unwanted side effects, such as deepening of the superior sulcus and fat atrophy; there was found to be enhancement of the eyelid crease in Asian eyelids and decrease in proptosis [20–23].

Oral ruxolitinib and other JAK inhibitors, such as tofacitinib, have been shown to aid in the treatment of alopecia areata, alopecia universalis, and alopecia totalis [24–30] as well as psoriasis [31].

Due to the above mentioned systemic side effects of oral ruxolitinib, topical ruxolitinib was developed recently. Topical ruxolitinib 0.6% cream twice a day has been proven to improve eyebrow and scalp hair growth [32].

## Conclusions

Oral ruxolitinib can aid in patients with madarosis or lack of eyelashes. Other JAK inhibitors may have a similar effect. This may aid patients with madarosis, refractory to the topical prostaglandins. Care should be taken with oral ruxolitinib, as the medication has side effects (Table 1). A review of all patients with MF treated with ruxolitinib to assess their facial and body hair may be warranted. Topical ruxolitinib cream may be useful; an ophthalmic drop or ointment may be beneficial, targeted therapy.

**Table 1:** Side effects of ruxolitinib

	Side effects	Treatment
Hematologic (dose-related)	Anemia, neutropenia, thrombocytopenia	CBC every 2–4 weeks
		Reduce dose
		Blood transfusion for anemia
Dermatologic	Bruising	
	Non-melanoma skin cancer (basal, squamous, Merkel)	Periodic skin examination
Neurologic	Progressive multifocal encephalopathy	Discontinue ruxolitinib
	Headache	
	Dizziness	
Endocrine	Hypercholesterolemia, elevated low-density lipoprotein, elevated triglycerides	Assess lipid panel 8–12 weeks after initiation
Gastrointestinal	Diarrhea	
Limbal	Edema (arm, leg, hand, feet)	
Immunologic	Tuberculosis	Manage tuberculosis
	Herpes zoster	Manage zoster
	Hepatitis B	Treat hepatitis

*CBC* complete blood count

## Abbreviations

AML: Acute myeomonocytic leukemia
BMB: Bone marrow biopsy
HES: Hypereosinophilic syndrome
Ig: Immunoglobulin
JAK: Janus kinase
MDS: Myelodysplastic syndrome
MF: Myelofibrosis
